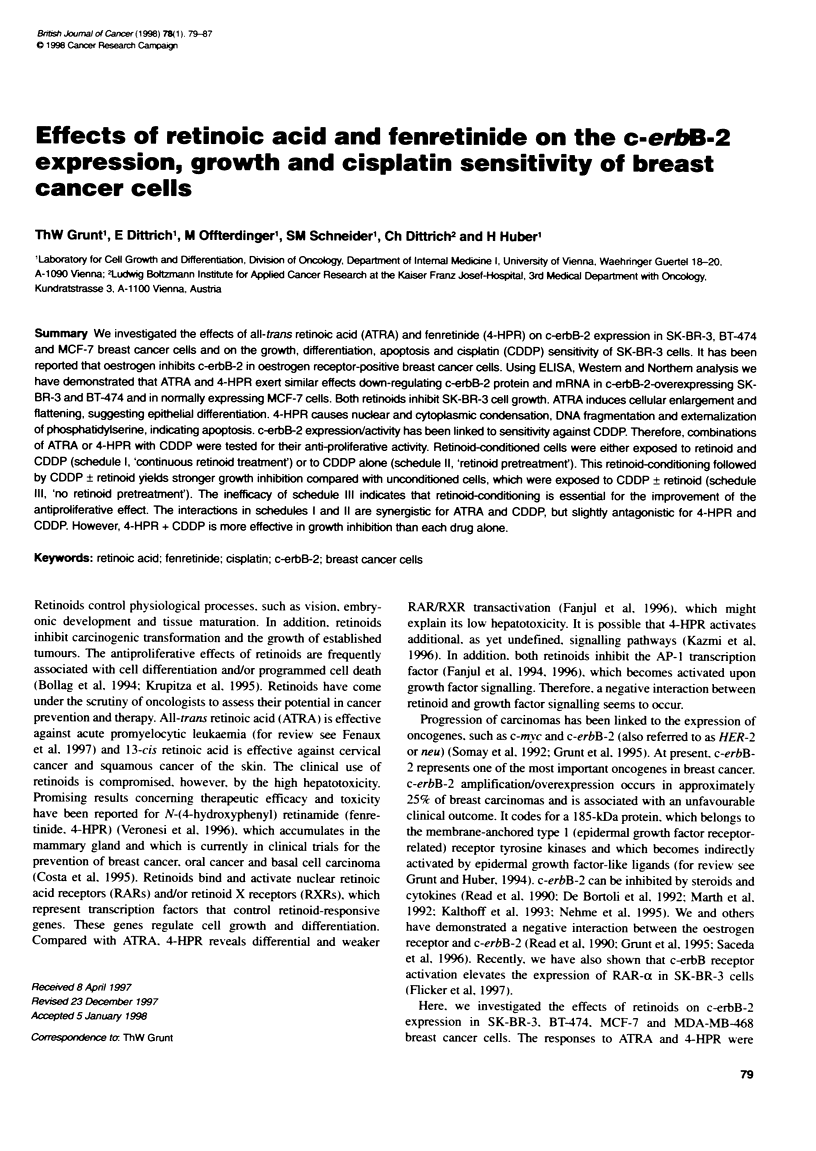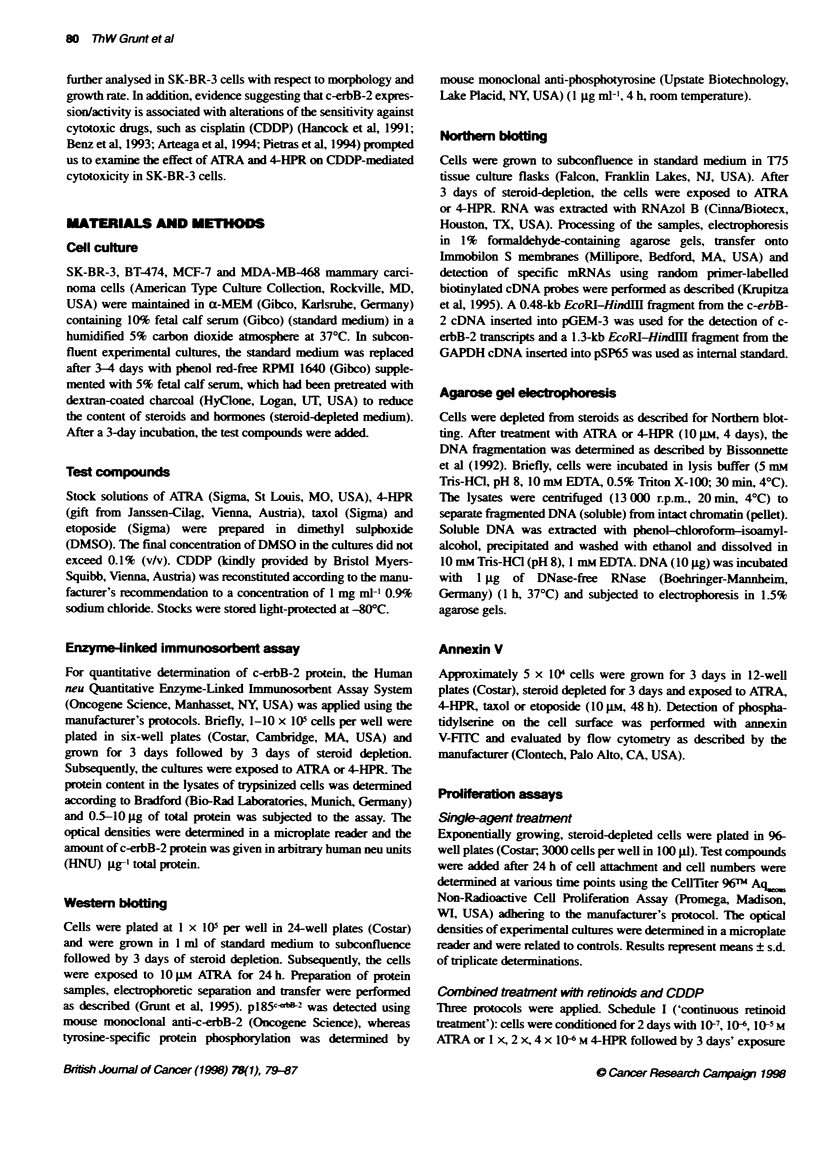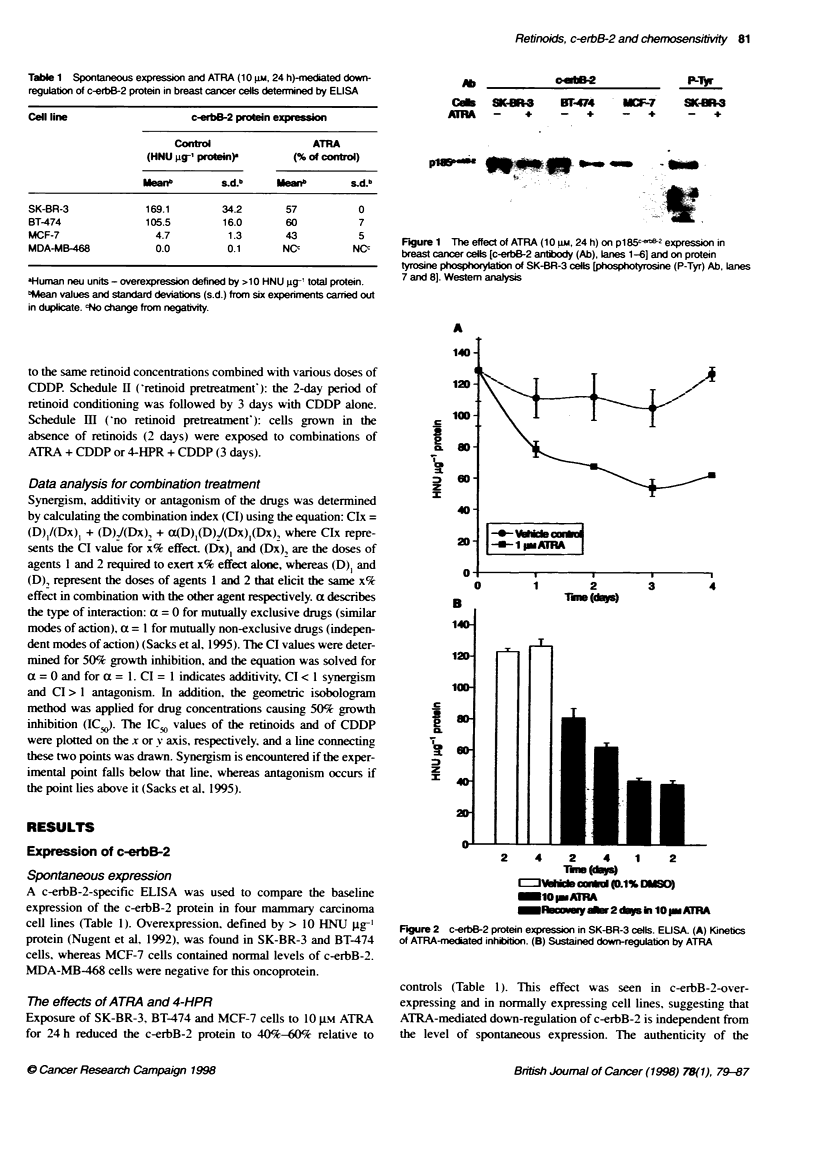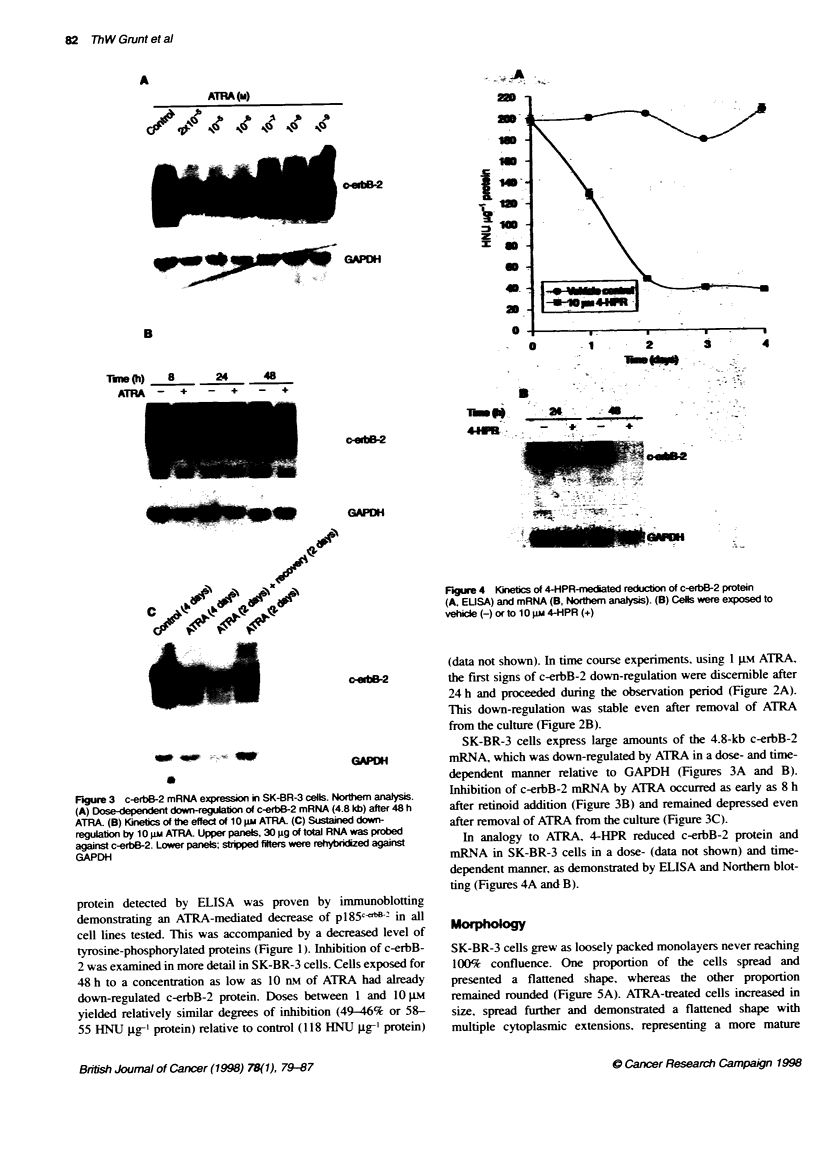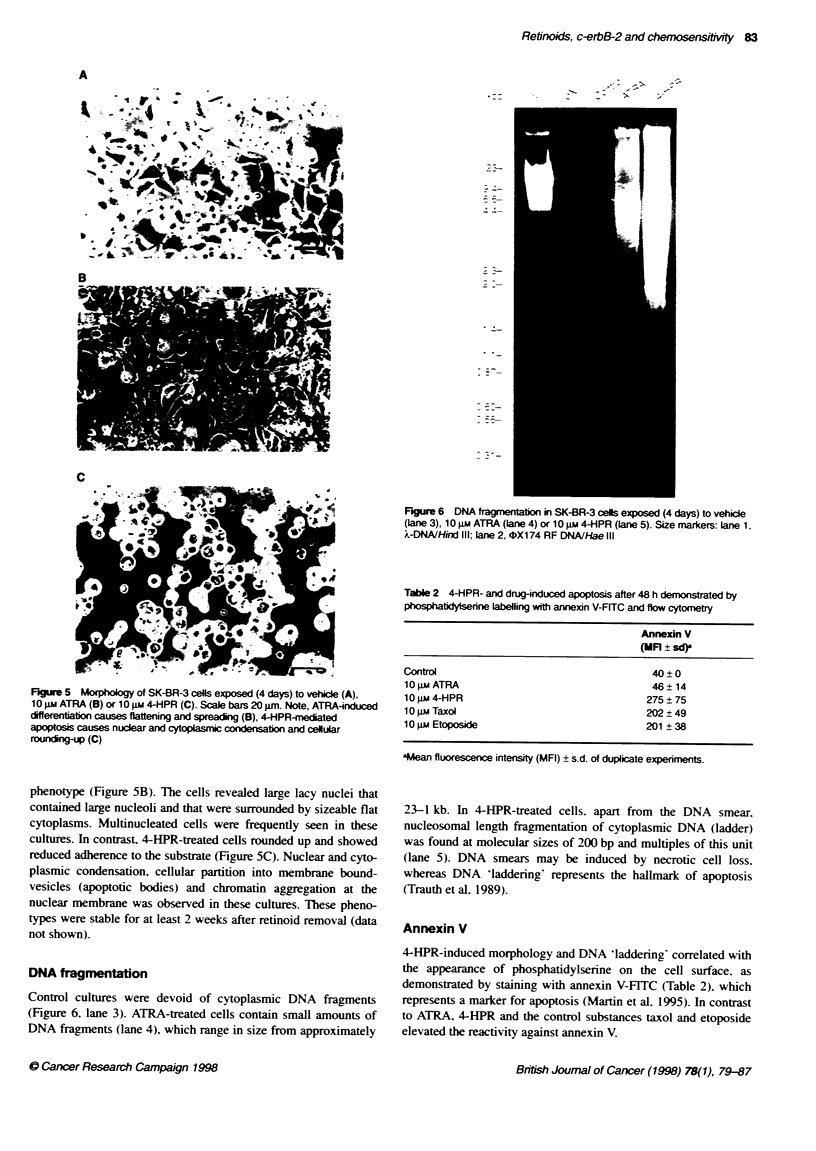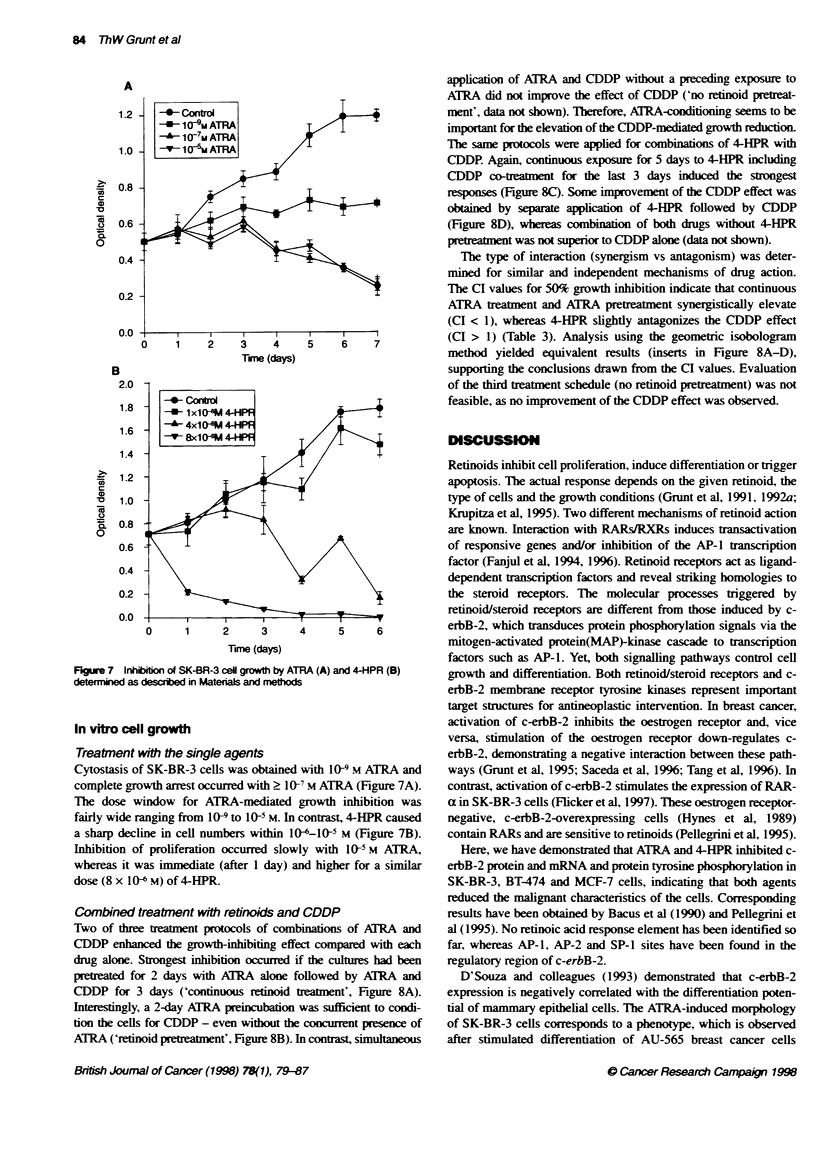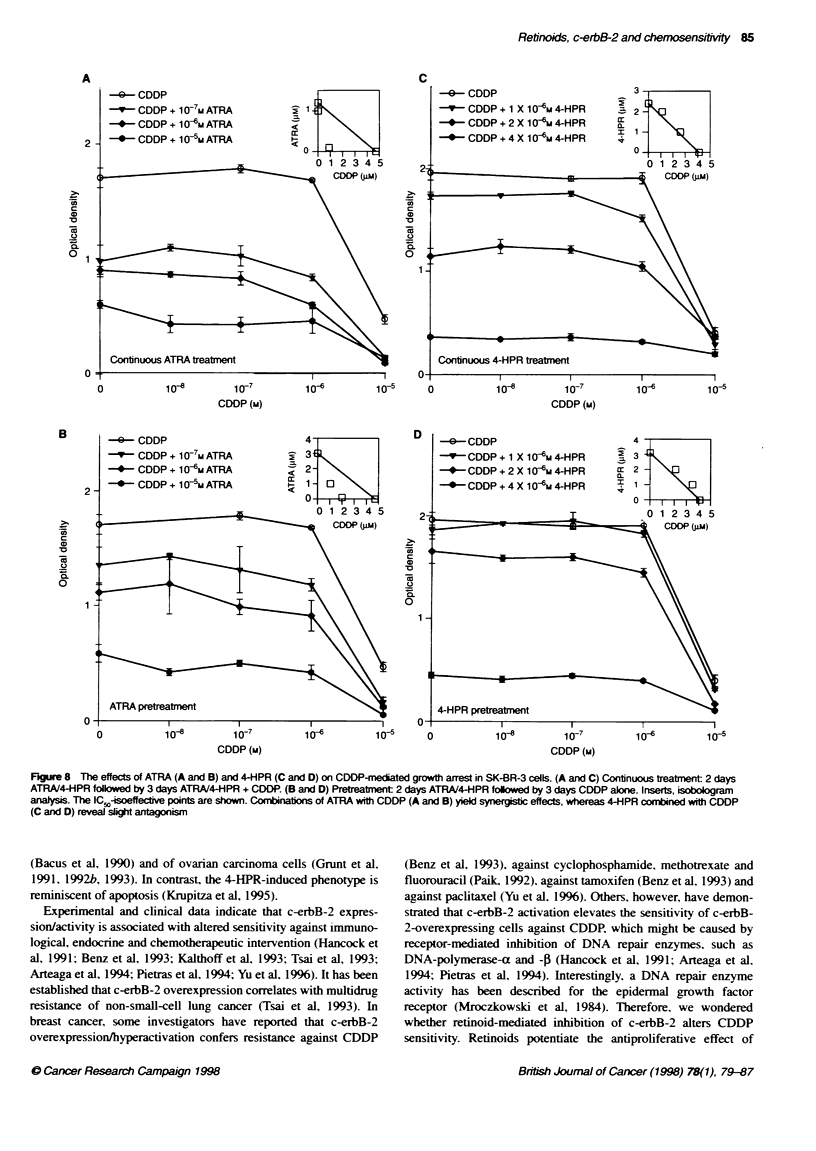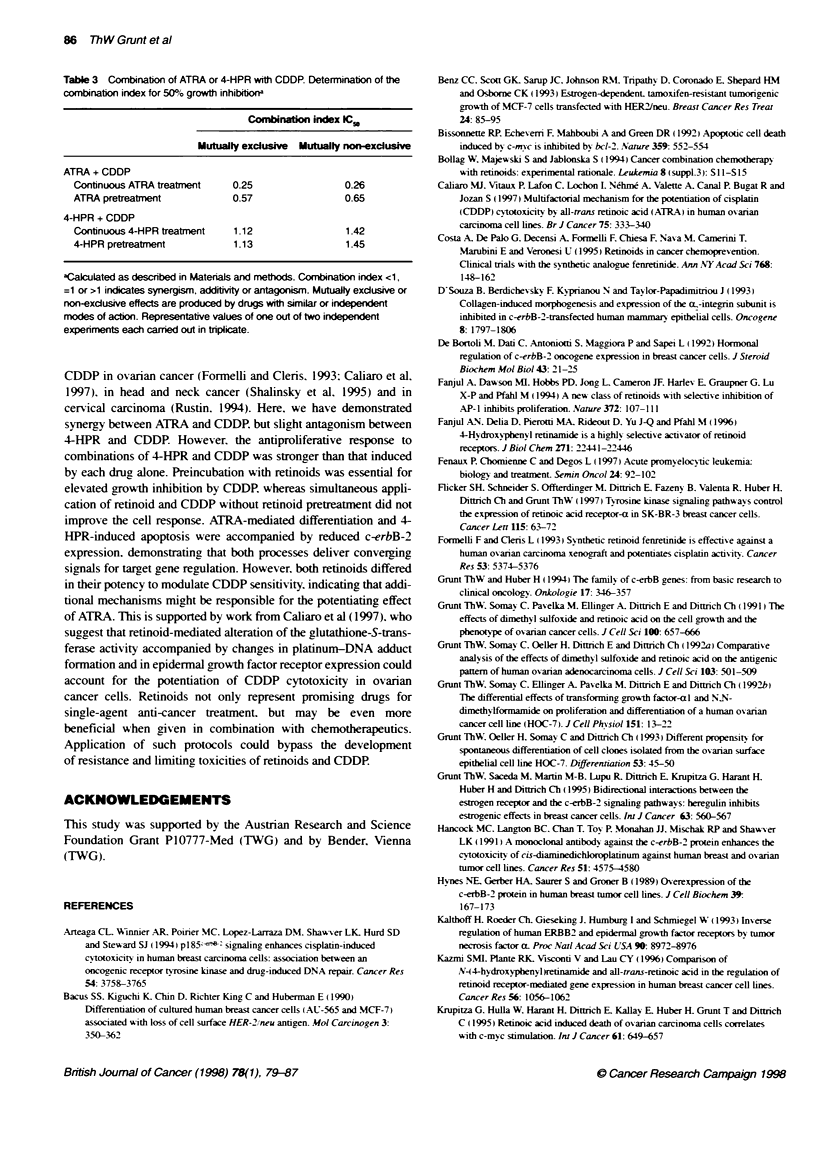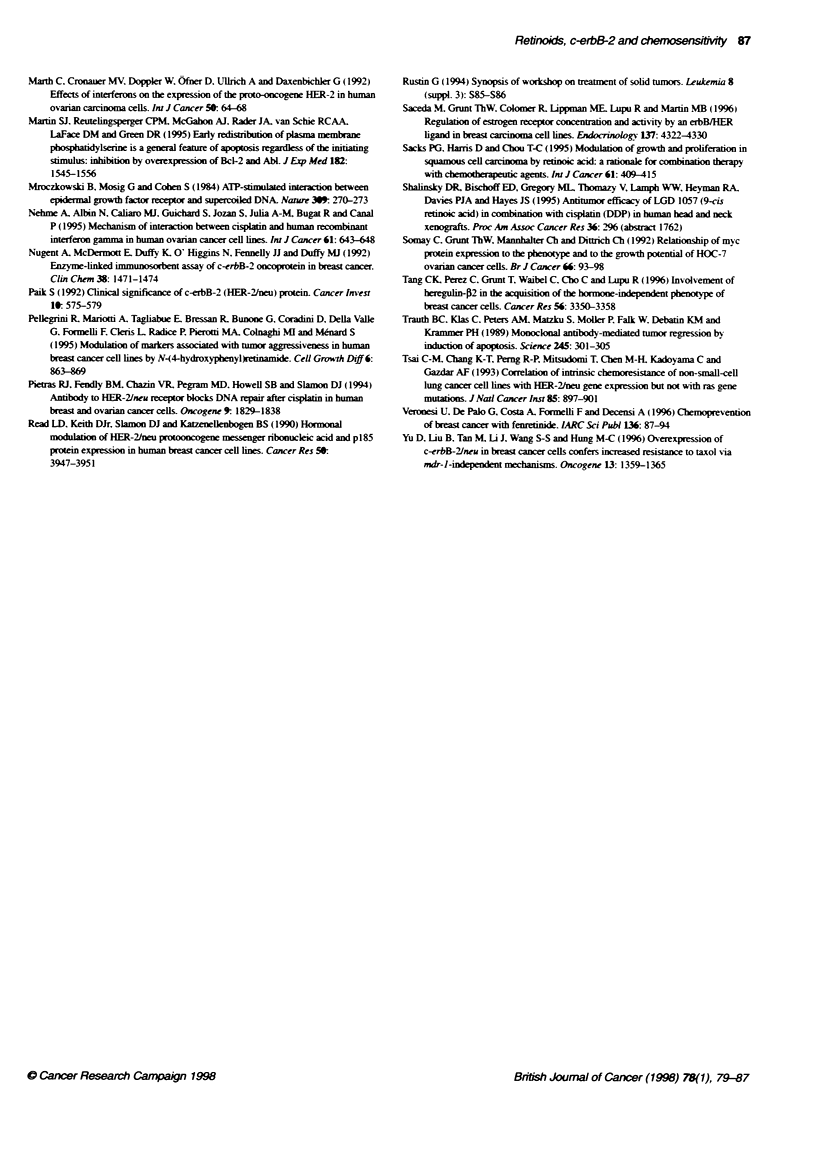# Effects of retinoic acid and fenretinide on the c-erbB-2 expression, growth and cisplatin sensitivity of breast cancer cells.

**DOI:** 10.1038/bjc.1998.446

**Published:** 1998-07

**Authors:** E. Dittrich, M. Offterdinger, S. M. Schneider, Ch Dittrich, H. Huber

**Affiliations:** Laboratory for Cell Growth and Differentiation, Department of Internal Medicine I, University of Vienna, Austria.

## Abstract

**Images:**


British Journal of Cancer (1998) 78(1), 79–87
© 1998 Cancer Research Campaign

### Effects of retinoic acid and fenretinide on the c-*erb*B-2 expression, growth and cisplatin sensitivity of breast cancer cells

**ThW Grunt[1], E Dittrich[1], M Offterdinger[1], SM Schneider[1], Ch Dittrich[2] and H Huber[1]**

[1]Laboratory for Cell Growth and Differentiation, Division of Oncology, Department of Internal Medicine I, University of Vienna, Waehringer Guertel 18–20, A-1090 Vienna; [2]Ludwig Boltzmann Institute for Applied Cancer Research at the Kaiser Franz Josef-Hospital, 3rd Medical Department with Oncology, Kundratstrasse 3, A-1100 Vienna, Austria

**Summary** We investigated the effects of all-*trans* retinoic acid (ATRA) and fenretinide (4-HPR) on c-erbB-2 expression in SK-BR-3, BT-474 and MCF-7 breast cancer cells and on the growth, differentiation, apoptosis and cisplatin (CDDP) sensitivity of SK-BR-3 cells. It has been reported that oestrogen inhibits c-erbB-2 in oestrogen receptor-positive breast cancer cells. Using ELISA, Western and Northern analysis we have demonstrated that ATRA and 4-HPR exert similar effects down-regulating c-erbB-2 protein and mRNA in c-erbB-2-overexpressing SK-BR-3 and BT-474 and in normally expressing MCF-7 cells. Both retinoids inhibit SK-BR-3 cell growth. ATRA induces cellular enlargement and flattening, suggesting epithelial differentiation. 4-HPR causes nuclear and cytoplasmic condensation, DNA fragmentation and externalization of phosphatidylserine, indicating apoptosis. c-erbB-2 expression/activity has been linked to sensitivity against CDDP. Therefore, combinations of ATRA or 4-HPR with CDDP were tested for their anti-proliferative activity. Retinoid-conditioned cells were either exposed to retinoid and CDDP (schedule I, 'continuous retinoid treatment') or to CDDP alone (schedule II, 'retinoid pretreatment'). This retinoid-conditioning followed by CDDP ± retinoid yields stronger growth inhibition compared with unconditioned cells, which were exposed to CDDP ± retinoid (schedule III, 'no retinoid pretreatment'). The inefficacy of schedule III indicates that retinoid-conditioning is essential for the improvement of the antiproliferative effect. The interactions in schedules I and II are synergistic for ATRA and CDDP, but slightly antagonistic for 4-HPR and CDDP. However, 4-HPR + CDDP is more effective in growth inhibition than each drug alone.

**Keywords:** retinoic acid; fenretinide; cisplatin; c-erbB-2; breast cancer cells

Retinoids control physiological processes, such as vision, embryonic development and tissue maturation. In addition, retinoids inhibit carcinogenic transformation and the growth of established tumours. The antiproliferative effects of retinoids are frequently associated with cell differentiation and/or programmed cell death (Bollag et al, 1994; Krupitza et al, 1995). Retinoids have come under the scrutiny of oncologists to assess their potential in cancer prevention and therapy. All-*trans* retinoic acid (ATRA) is effective against acute promyelocytic leukaemia (for review see Fenaux et al, 1997) and 13-*cis* retinoic acid is effective against cervical cancer and squamous cancer of the skin. The clinical use of retinoids is compromised, however, by the high hepatotoxicity. Promising results concerning therapeutic efficacy and toxicity have been reported for *N*-(4-hydroxyphenyl) retinamide (fenretinide, 4-HPR) (Veronesi et al, 1996), which accumulates in the mammary gland and which is currently in clinical trials for the prevention of breast cancer, oral cancer and basal cell carcinoma (Costa et al, 1995). Retinoids bind and activate nuclear retinoic acid receptors (RARs) and/or retinoid X receptors (RXRs), which represent transcription factors that control retinoid-responsive genes. These genes regulate cell growth and differentiation. Compared with ATRA, 4-HPR reveals differential and weaker RAR/RXR transactivation (Fanjul et al, 1996), which might explain its low hepatotoxicity. It is possible that 4-HPR activates additional, as yet undefined, signalling pathways (Kazmi et al, 1996). In addition, both retinoids inhibit the AP-1 transcription factor (Fanjul et al, 1994, 1996), which becomes activated upon growth factor signalling. Therefore, a negative interaction between retinoid and growth factor signalling seems to occur.

Progression of carcinomas has been linked to the expression of oncogenes, such as c-*myc* and c-*erb*B-2 (also referred to as *HER*-2 or *neu*) (Somay et al, 1992; Grunt et al, 1995). At present, c-*erb*B-2 represents one of the most important oncogenes in breast cancer. c-*erb*B-2 amplification/overexpression occurs in approximately 25% of breast carcinomas and is associated with an unfavourable clinical outcome. It codes for a 185-kDa protein, which belongs to the membrane-anchored type 1 (epidermal growth factor receptor-related) receptor tyrosine kinases and which becomes indirectly activated by epidermal growth factor-like ligands (for review see Grunt and Huber, 1994). c-*erb*B-2 can be inhibited by steroids and cytokines (Read et al, 1990; De Bortoli et al, 1992; Marth et al, 1992; Kalthoff et al, 1993; Nehme et al, 1995). We and others have demonstrated a negative interaction between the oestrogen receptor and c-*erb*B-2 (Read et al, 1990; Grunt et al, 1995; Saceda et al, 1996). Recently, we have also shown that c-erbB receptor activation elevates the expression of RAR-α in SK-BR-3 cells (Flicker et al, 1997).

Here, we investigated the effects of retinoids on c-erbB-2 expression in SK-BR-3, BT-474, MCF-7 and MDA-MB-468 breast cancer cells. The responses to ATRA and 4-HPR were

*Received 8 April 1997*
*Revised 23 December 1997*
*Accepted 5 January 1998*

*Correspondence to:* ThW Grunt further analysed in SK-BR-3 cells with respect to morphology and growth rate. In addition, evidence suggesting that c-erbB-2 expression/activity is associated with alterations of the sensitivity against cytotoxic drugs, such as cisplatin (CDDP) (Hancock et al, 1991; Benz et al, 1993; Arteaga et al, 1994; Pietras et al, 1994) prompted us to examine the effect of ATRA and 4-HPR on CDDP-mediated cytotoxicity in SK-BR-3 cells.

### MATERIALS AND METHODS

### Cell culture

SK-BR-3, BT-474, MCF-7 and MDA-MB-468 mammary carcinoma cells (American Type Culture Collection, Rockville, MD, USA) were maintained in α-MEM (Gibco, Karlsruhe, Germany) containing 10% fetal calf serum (Gibco) (standard medium) in a humidified 5% carbon dioxide atmosphere at 37°C. In subconfluent experimental cultures, the standard medium was replaced after 3–4 days with phenol red-free RPMI 1640 (Gibco) supplemented with 5% fetal calf serum, which had been pretreated with dextran-coated charcoal (HyClone, Logan, UT, USA) to reduce the content of steroids and hormones (steroid-depleted medium). After a 3-day incubation, the test compounds were added.

### Test compounds

Stock solutions of ATRA (Sigma, St Louis, MO, USA), 4-HPR (gift from Janssen-Cilag, Vienna, Austria), taxol (Sigma) and etoposide (Sigma) were prepared in dimethyl sulphoxide (DMSO). The final concentration of DMSO in the cultures did not exceed 0.1% (v/v). CDDP (kindly provided by Bristol Myers-Squibb, Vienna, Austria) was reconstituted according to the manufacturer's recommendation to a concentration of 1 mg $ml^{-1}$ 0.9% sodium chloride. Stocks were stored light-protected at –80°C.

### Enzyme-linked immunosorbent assay

For quantitative determination of c-erbB-2 protein, the Human *neu* Quantitative Enzyme-Linked Immunosorbent Assay System (Oncogene Science, Manhasset, NY, USA) was applied using the manufacturer's protocols. Briefly, 1–10 × $10^5$ cells per well were plated in six-well plates (Costar, Cambridge, MA, USA) and grown for 3 days followed by 3 days of steroid depletion. Subsequently, the cultures were exposed to ATRA or 4-HPR. The protein content in the lysates of trypsinized cells was determined according to Bradford (Bio-Rad Laboratories, Munich, Germany) and 0.5–10 μg of total protein was subjected to the assay. The optical densities were determined in a microplate reader and the amount of c-erbB-2 protein was given in arbitrary human neu units (HNU) $\mu g^{-1}$ total protein.

### Western blotting

Cells were plated at 1 × $10^5$ per well in 24-well plates (Costar) and were grown in 1 ml of standard medium to subconfluence followed by 3 days of steroid depletion. Subsequently, the cells were exposed to 10 μM ATRA for 24 h. Preparation of protein samples, electrophoretic separation and transfer were performed as described (Grunt et al, 1995). $p185^{c\text{-}erbB\text{-}2}$ was detected using mouse monoclonal anti-c-erbB-2 (Oncogene Science), whereas tyrosine-specific protein phosphorylation was determined by mouse monoclonal anti-phosphotyrosine (Upstate Biotechnology, Lake Placid, NY, USA) (1 μg $ml^{-1}$, 4 h, room temperature).

### Northern blotting

Cells were grown to subconfluence in standard medium in T75 tissue culture flasks (Falcon, Franklin Lakes, NJ, USA). After 3 days of steroid-depletion, the cells were exposed to ATRA or 4-HPR. RNA was extracted with RNAzol B (Cinna/Biotecx, Houston, TX, USA). Processing of the samples, electrophoresis in 1% formaldehyde-containing agarose gels, transfer onto Immobilon S membranes (Millipore, Bedford, MA, USA) and detection of specific mRNAs using random primer-labelled biotinylated cDNA probes were performed as described (Krupitza et al, 1995). A 0.48-kb *Eco*RI–*Hin*dIII fragment from the c-*erb*B-2 cDNA inserted into pGEM-3 was used for the detection of c-erbB-2 transcripts and a 1.3-kb *Eco*RI–*Hin*dIII fragment from the GAPDH cDNA inserted into pSP65 was used as internal standard.

### Agarose gel electrophoresis

Cells were depleted from steroids as described for Northern blotting. After treatment with ATRA or 4-HPR (10 μM, 4 days), the DNA fragmentation was determined as described by Bissonnette et al (1992). Briefly, cells were incubated in lysis buffer (5 mM Tris-HCl, pH 8, 10 mM EDTA, 0.5% Triton X-100; 30 min, 4°C). The lysates were centrifuged (13 000 r.p.m., 20 min, 4°C) to separate fragmented DNA (soluble) from intact chromatin (pellet). Soluble DNA was extracted with phenol–chloroform–isoamyl-alcohol, precipitated and washed with ethanol and dissolved in 10 mM Tris-HCl (pH 8), 1 mM EDTA. DNA (10 μg) was incubated with 1 μg of DNase-free RNase (Boehringer-Mannheim, Germany) (1 h, 37°C) and subjected to electrophoresis in 1.5% agarose gels.

### Annexin V

Approximately 5 × $10^4$ cells were grown for 3 days in 12-well plates (Costar), steroid depleted for 3 days and exposed to ATRA, 4-HPR, taxol or etoposide (10 μM, 48 h). Detection of phosphatidylserine on the cell surface was performed with annexin V-FITC and evaluated by flow cytometry as described by the manufacturer (Clontech, Palo Alto, CA, USA).

### Proliferation assays

*Single-agent treatment*

Exponentially growing, steroid-depleted cells were plated in 96-well plates (Costar; 3000 cells per well in 100 μl). Test compounds were added after 24 h of cell attachment and cell numbers were determined at various time points using the CellTiter 96™ $Aq_{ueous}$ Non-Radioactive Cell Proliferation Assay (Promega, Madison, WI, USA) adhering to the manufacturer's protocol. The optical densities of experimental cultures were determined in a microplate reader and were related to controls. Results represent means ± s.d. of triplicate determinations.

*Combined treatment with retinoids and CDDP*

Three protocols were applied. Schedule I ('continuous retinoid treatment'): cells were conditioned for 2 days with $10^{-7}$, $10^{-6}$, $10^{-5}$ M ATRA or 1 ×, 2 ×, 4 × $10^{-6}$ M 4-HPR followed by 3 days' exposure

© Cancer Research Campaign 1998

**Table 1** Spontaneous expression and ATRA (10 μM, 24 h)-mediated down-regulation of c-erbB-2 protein in breast cancer cells determined by ELISA

| **Cell line** | **c-erbB-2 protein expression** | | | |
|---|---|---|---|---|
| | **Control (HNU μg⁻¹ protein)[a]** | | **ATRA (% of control)** | |
| | **Mean[b]** | **s.d.[b]** | **Mean[b]** | **s.d.[b]** |
| SK-BR-3 | 169.1 | 34.2 | 57 | 0 |
| BT-474 | 105.5 | 16.0 | 60 | 7 |
| MCF-7 | 4.7 | 1.3 | 43 | 5 |
| MDA-MB-468 | 0.0 | 0.1 | NC[c] | NC[c] |

[a]Human neu units – overexpression defined by >10 HNU $\mu g^{-1}$ total protein. [b]Mean values and standard deviations (s.d.) from six experiments carried out in duplicate. [c]No change from negativity.

to the same retinoid concentrations combined with various doses of CDDP. Schedule II ('retinoid pretreatment'): the 2-day period of retinoid conditioning was followed by 3 days with CDDP alone. Schedule III ('no retinoid pretreatment'): cells grown in the absence of retinoids (2 days) were exposed to combinations of ATRA + CDDP or 4-HPR + CDDP (3 days).

### *Data analysis for combination treatment*

Synergism, additivity or antagonism of the drugs was determined by calculating the combination index (CI) using the equation: $CIx = (D)_1/(Dx)_1 + (D)_2/(Dx)_2 + \alpha(D)_1(D)_2/(Dx)_1(Dx)_2$ where CIx represents the CI value for x% effect. $(Dx)_1$ and $(Dx)_2$ are the doses of agents 1 and 2 required to exert x% effect alone, whereas $(D)_1$ and $(D)_2$ represent the doses of agents 1 and 2 that elicit the same x% effect in combination with the other agent respectively. $\alpha$ describes the type of interaction: $\alpha = 0$ for mutually exclusive drugs (similar modes of action), $\alpha = 1$ for mutually non-exclusive drugs (independent modes of action) (Sacks et al, 1995). The CI values were determined for 50% growth inhibition, and the equation was solved for $\alpha = 0$ and for $\alpha = 1$. CI = 1 indicates additivity, CI < 1 synergism and CI > 1 antagonism. In addition, the geometric isobologram method was applied for drug concentrations causing 50% growth inhibition ($IC_{50}$). The $IC_{50}$ values of the retinoids and of CDDP were plotted on the $x$ or $y$ axis, respectively, and a line connecting these two points was drawn. Synergism is encountered if the experimental point falls below that line, whereas antagonism occurs if the point lies above it (Sacks et al, 1995).

### RESULTS

### Expression of c-erbB-2

#### *Spontaneous expression*

A c-erbB-2-specific ELISA was used to compare the baseline expression of the c-erbB-2 protein in four mammary carcinoma cell lines (Table 1). Overexpression, defined by > 10 HNU $\mu g^{-1}$ protein (Nugent et al, 1992), was found in SK-BR-3 and BT-474 cells, whereas MCF-7 cells contained normal levels of c-erbB-2. MDA-MB-468 cells were negative for this oncoprotein.

#### *The effects of ATRA and 4-HPR*

Exposure of SK-BR-3, BT-474 and MCF-7 cells to 10 μM ATRA for 24 h reduced the c-erbB-2 protein to 40%–60% relative to

**Figure 1** The effect of ATRA (10 μM, 24 h) on p185[c-erbB-2] expression in breast cancer cells [c-erbB-2 antibody (Ab), lanes 1–6] and on protein tyrosine phosphorylation of SK-BR-3 cells [phosphotyrosine (P-Tyr) Ab, lanes 7 and 8]. Western analysis

**Figure 2** c-erbB-2 protein expression in SK-BR-3 cells. ELISA. (**A**) Kinetics of ATRA-mediated inhibition. (**B**) Sustained down-regulation by ATRA

controls (Table 1). This effect was seen in c-erbB-2-over-expressing and in normally expressing cell lines, suggesting that ATRA-mediated down-regulation of c-erbB-2 is independent from the level of spontaneous expression. The authenticity of the

© Cancer Research Campaign 1998 

**Figure 3** c-erbB-2 mRNA expression in SK-BR-3 cells. Northern analysis. (**A**) Dose-dependent down-regulation of c-erbB-2 mRNA (4.8 kb) after 48 h ATRA. (**B**) Kinetics of the effect of 10 μM ATRA. (**C**) Sustained down-regulation by 10 μM ATRA. Upper panels, 30 μg of total RNA was probed against c-erbB-2. Lower panels; stripped filters were rehybridized against GAPDH

**Figure 4** Kinetics of 4-HPR-mediated reduction of c-erbB-2 protein (**A**, ELISA) and mRNA (**B**, Northern analysis). (**B**) Cells were exposed to vehicle (–) or to 10 μM 4-HPR (+)

protein detected by ELISA was proven by immunoblotting demonstrating an ATRA-mediated decrease of $p185^{c\text{-}erbB\text{-}2}$ in all cell lines tested. This was accompanied by a decreased level of tyrosine-phosphorylated proteins (Figure 1). Inhibition of c-erbB-2 was examined in more detail in SK-BR-3 cells. Cells exposed for 48 h to a concentration as low as 10 nM of ATRA had already down-regulated c-erbB-2 protein. Doses between 1 and 10 μM yielded relatively similar degrees of inhibition (49–46% or 58–55 HNU $\mu g^{-1}$ protein) relative to control (118 HNU $\mu g^{-1}$ protein) (data not shown). In time course experiments, using 1 μM ATRA, the first signs of c-erbB-2 down-regulation were discernible after 24 h and proceeded during the observation period (Figure 2A). This down-regulation was stable even after removal of ATRA from the culture (Figure 2B).

SK-BR-3 cells express large amounts of the 4.8-kb c-erbB-2 mRNA, which was down-regulated by ATRA in a dose- and time-dependent manner relative to GAPDH (Figures 3A and B). Inhibition of c-erbB-2 mRNA by ATRA occurred as early as 8 h after retinoid addition (Figure 3B) and remained depressed even after removal of ATRA from the culture (Figure 3C).

In analogy to ATRA, 4-HPR reduced c-erbB-2 protein and mRNA in SK-BR-3 cells in a dose- (data not shown) and time-dependent manner, as demonstrated by ELISA and Northern blotting (Figures 4A and B).

### Morphology

SK-BR-3 cells grew as loosely packed monolayers never reaching 100% confluence. One proportion of the cells spread and presented a flattened shape, whereas the other proportion remained rounded (Figure 5A). ATRA-treated cells increased in size, spread further and demonstrated a flattened shape with multiple cytoplasmic extensions, representing a more mature

© Cancer Research Campaign 1998

**Figure 5** Morphology of SK-BR-3 cells exposed (4 days) to vehicle (**A**), 10 μM ATRA (**B**) or 10 μM 4-HPR (**C**). Scale bars 20 μm. Note, ATRA-induced differentiation causes flattening and spreading (**B**), 4-HPR-mediated apoptosis causes nuclear and cytoplasmic condensation and cellular rounding-up (**C**)

**Figure 6** DNA fragmentation in SK-BR-3 cells exposed (4 days) to vehicle (lane 3), 10 μM ATRA (lane 4) or 10 μM 4-HPR (lane 5). Size markers: lane 1, λ-DNA/*Hind* III; lane 2, ΦX174 RF DNA/*Hae* III

**Table 2** 4-HPR- and drug-induced apoptosis after 48 h demonstrated by phosphatidylserine labelling with annexin V-FITC and flow cytometry

| | **Annexin V (MFI ± sd)[a]** |
|---|---|
| Control | 40 ± 0 |
| 10 μM ATRA | 46 ± 14 |
| 10 μM 4-HPR | 275 ± 75 |
| 10 μM Taxol | 202 ± 49 |
| 10 μM Etoposide | 201 ± 38 |

[a]Mean fluorescence intensity (MFI) ± s.d. of duplicate experiments.

phenotype (Figure 5B). The cells revealed large lacy nuclei that contained large nucleoli and that were surrounded by sizeable flat cytoplasms. Multinucleated cells were frequently seen in these cultures. In contrast, 4-HPR-treated cells rounded up and showed reduced adherence to the substrate (Figure 5C). Nuclear and cytoplasmic condensation, cellular partition into membrane bound-vesicles (apoptotic bodies) and chromatin aggregation at the nuclear membrane was observed in these cultures. These phenotypes were stable for at least 2 weeks after retinoid removal (data not shown).

### DNA fragmentation

Control cultures were devoid of cytoplasmic DNA fragments (Figure 6, lane 3). ATRA-treated cells contain small amounts of DNA fragments (lane 4), which range in size from approximately 23–1 kb. In 4-HPR-treated cells, apart from the DNA smear, nucleosomal length fragmentation of cytoplasmic DNA (ladder) was found at molecular sizes of 200 bp and multiples of this unit (lane 5). DNA smears may be induced by necrotic cell loss, whereas DNA 'laddering' represents the hallmark of apoptosis (Trauth et al, 1989).

### Annexin V

4-HPR-induced morphology and DNA 'laddering' correlated with the appearance of phosphatidylserine on the cell surface, as demonstrated by staining with annexin V-FITC (Table 2), which represents a marker for apoptosis (Martin et al, 1995). In contrast to ATRA, 4-HPR and the control substances taxol and etoposide elevated the reactivity against annexin V.

© Cancer Research Campaign 1998 

**Figure 7** Inhibition of SK-BR-3 cell growth by ATRA (**A**) and 4-HPR (**B**) determined as described in Materials and methods

### In vitro cell growth

### *Treatment with the single agents*

Cytostasis of SK-BR-3 cells was obtained with $10^{-9}$ M ATRA and complete growth arrest occurred with $\geq 10^{-7}$ M ATRA (Figure 7A). The dose window for ATRA-mediated growth inhibition was fairly wide ranging from $10^{-9}$ to $10^{-5}$ M. In contrast, 4-HPR caused a sharp decline in cell numbers within $10^{-6}$–$10^{-5}$ M (Figure 7B). Inhibition of proliferation occurred slowly with $10^{-5}$ M ATRA, whereas it was immediate (after 1 day) and higher for a similar dose ($8 \times 10^{-6}$ M) of 4-HPR.

### *Combined treatment with retinoids and CDDP*

Two of three treatment protocols of combinations of ATRA and CDDP enhanced the growth-inhibiting effect compared with each drug alone. Strongest inhibition occurred if the cultures had been pretreated for 2 days with ATRA alone followed by ATRA and CDDP for 3 days ('continuous retinoid treatment', Figure 8A). Interestingly, a 2-day ATRA preincubation was sufficient to condition the cells for CDDP – even without the concurrent presence of ATRA ('retinoid pretreatment', Figure 8B). In contrast, simultaneous application of ATRA and CDDP without a preceding exposure to ATRA did not improve the effect of CDDP ('no retinoid pretreatment', data not shown). Therefore, ATRA-conditioning seems to be important for the elevation of the CDDP-mediated growth reduction. The same protocols were applied for combinations of 4-HPR with CDDP. Again, continuous exposure for 5 days to 4-HPR including CDDP co-treatment for the last 3 days induced the strongest responses (Figure 8C). Some improvement of the CDDP effect was obtained by separate application of 4-HPR followed by CDDP (Figure 8D), whereas combination of both drugs without 4-HPR pretreatment was not superior to CDDP alone (data not shown).

The type of interaction (synergism vs antagonism) was determined for similar and independent mechanisms of drug action. The CI values for 50% growth inhibition indicate that continuous ATRA treatment and ATRA pretreatment synergistically elevate (CI < 1), whereas 4-HPR slightly antagonizes the CDDP effect (CI > 1) (Table 3). Analysis using the geometric isobologram method yielded equivalent results (inserts in Figure 8A–D), supporting the conclusions drawn from the CI values. Evaluation of the third treatment schedule (no retinoid pretreatment) was not feasible, as no improvement of the CDDP effect was observed.

### DISCUSSION

Retinoids inhibit cell proliferation, induce differentiation or trigger apoptosis. The actual response depends on the given retinoid, the type of cells and the growth conditions (Grunt et al, 1991, 1992*a*; Krupitza et al, 1995). Two different mechanisms of retinoid action are known. Interaction with RARs/RXRs induces transactivation of responsive genes and/or inhibition of the AP-1 transcription factor (Fanjul et al, 1994, 1996). Retinoid receptors act as ligand-dependent transcription factors and reveal striking homologies to the steroid receptors. The molecular processes triggered by retinoid/steroid receptors are different from those induced by c-erbB-2, which transduces protein phosphorylation signals via the mitogen-activated protein(MAP)-kinase cascade to transcription factors such as AP-1. Yet, both signalling pathways control cell growth and differentiation. Both retinoid/steroid receptors and c-erbB-2 membrane receptor tyrosine kinases represent important target structures for antineoplastic intervention. In breast cancer, activation of c-erbB-2 inhibits the oestrogen receptor and, vice versa, stimulation of the oestrogen receptor down-regulates c-erbB-2, demonstrating a negative interaction between these pathways (Grunt et al, 1995; Saceda et al, 1996; Tang et al, 1996). In contrast, activation of c-erbB-2 stimulates the expression of RAR-α in SK-BR-3 cells (Flicker et al, 1997). These oestrogen receptor-negative, c-erbB-2-overexpressing cells (Hynes et al, 1989) contain RARs and are sensitive to retinoids (Pellegrini et al, 1995).

Here, we have demonstrated that ATRA and 4-HPR inhibited c-erbB-2 protein and mRNA and protein tyrosine phosphorylation in SK-BR-3, BT-474 and MCF-7 cells, indicating that both agents reduced the malignant characteristics of the cells. Corresponding results have been obtained by Bacus et al (1990) and Pellegrini et al (1995). No retinoic acid response element has been identified so far, whereas AP-1, AP-2 and SP-1 sites have been found in the regulatory region of c-*erb*B-2.

D'Souza and colleagues (1993) demonstrated that c-erbB-2 expression is negatively correlated with the differentiation potential of mammary epithelial cells. The ATRA-induced morphology of SK-BR-3 cells corresponds to a phenotype, which is observed after stimulated differentiation of AU-565 breast cancer cells

 © Cancer Research Campaign 1998

**Figure 8** The effects of ATRA (**A** and **B**) and 4-HPR (**C** and **D**) on CDDP-mediated growth arrest in SK-BR-3 cells. (**A** and **C**) Continuous treatment: 2 days ATRA/4-HPR followed by 3 days ATRA/4-HPR + CDDP. (**B** and **D**) Pretreatment: 2 days ATRA/4-HPR followed by 3 days CDDP alone. Inserts, isobologram analysis. The $IC_{50}$-isoeffective points are shown. Combinations of ATRA with CDDP (**A** and **B**) yield synergistic effects, whereas 4-HPR combined with CDDP (**C** and **D**) reveal slight antagonism

(Bacus et al, 1990) and of ovarian carcinoma cells (Grunt et al, 1991, 1992*b*, 1993). In contrast, the 4-HPR-induced phenotype is reminiscent of apoptosis (Krupitza et al, 1995).

Experimental and clinical data indicate that c-erbB-2 expression/activity is associated with altered sensitivity against immunological, endocrine and chemotherapeutic intervention (Hancock et al, 1991; Benz et al, 1993; Kalthoff et al, 1993; Tsai et al, 1993; Arteaga et al, 1994; Pietras et al, 1994; Yu et al, 1996). It has been established that c-erbB-2 overexpression correlates with multidrug resistance of non-small-cell lung cancer (Tsai et al, 1993). In breast cancer, some investigators have reported that c-erbB-2 overexpression/hyperactivation confers resistance against CDDP (Benz et al, 1993), against cyclophosphamide, methotrexate and fluorouracil (Paik, 1992), against tamoxifen (Benz et al, 1993) and against paclitaxel (Yu et al, 1996). Others, however, have demonstrated that c-erbB-2 activation elevates the sensitivity of c-erbB-2-overexpressing cells against CDDP, which might be caused by receptor-mediated inhibition of DNA repair enzymes, such as DNA-polymerase-α and -β (Hancock et al, 1991; Arteaga et al, 1994; Pietras et al, 1994). Interestingly, a DNA repair enzyme activity has been described for the epidermal growth factor receptor (Mroczkowski et al, 1984). Therefore, we wondered whether retinoid-mediated inhibition of c-erbB-2 alters CDDP sensitivity. Retinoids potentiate the antiproliferative effect of

**Table 3** Combination of ATRA or 4-HPR with CDDP. Determination of the combination index for 50% growth inhibition[a]

| | Combination index $IC_{50}$ | |
|---|---|---|
| | **Mutually exclusive** | **Mutually non-exclusive** |
| ATRA + CDDP | | |
| Continuous ATRA treatment | 0.25 | 0.26 |
| ATRA pretreatment | 0.57 | 0.65 |
| 4-HPR + CDDP | | |
| Continuous 4-HPR treatment | 1.12 | 1.42 |
| 4-HPR pretreatment | 1.13 | 1.45 |

[a]Calculated as described in Materials and methods. Combination index <1, =1 or >1 indicates synergism, additivity or antagonism. Mutually exclusive or non-exclusive effects are produced by drugs with similar or independent modes of action. Representative values of one out of two independent experiments each carried out in triplicate.

CDDP in ovarian cancer (Formelli and Cleris, 1993; Caliaro et al, 1997), in head and neck cancer (Shalinsky et al, 1995) and in cervical carcinoma (Rustin, 1994). Here, we have demonstrated synergy between ATRA and CDDP, but slight antagonism between 4-HPR and CDDP. However, the antiproliferative response to combinations of 4-HPR and CDDP was stronger than that induced by each drug alone. Preincubation with retinoids was essential for elevated growth inhibition by CDDP, whereas simultaneous application of retinoid and CDDP without retinoid pretreatment did not improve the cell response. ATRA-mediated differentiation and 4-HPR-induced apoptosis were accompanied by reduced c-*erb*B-2 expression, demonstrating that both processes deliver converging signals for target gene regulation. However, both retinoids differed in their potency to modulate CDDP sensitivity, indicating that additional mechanisms might be responsible for the potentiating effect of ATRA. This is supported by work from Caliaro et al (1997), who suggest that retinoid-mediated alteration of the glutathione-*S*-transferase activity accompanied by changes in platinum–DNA adduct formation and in epidermal growth factor receptor expression could account for the potentiation of CDDP cytotoxicity in ovarian cancer cells. Retinoids not only represent promising drugs for single-agent anti-cancer treatment, but may be even more beneficial when given in combination with chemotherapeutics. Application of such protocols could bypass the development of resistance and limiting toxicities of retinoids and CDDP.

### ACKNOWLEDGEMENTS

This study was supported by the Austrian Research and Science Foundation Grant P10777-Med (TWG) and by Bender, Vienna (TWG).

### REFERENCES

Arteaga CL, Winnier AR, Poirier MC, Lopez-Larraza DM, Shawver LK, Hurd SD and Steward SJ (1994) p185[c-erbB-2] signaling enhances cisplatin-induced cytotoxicity in human breast carcinoma cells: association between an oncogenic receptor tyrosine kinase and drug-induced DNA repair. *Cancer Res* **54**: 3758–3765

Bacus SS, Kiguchi K, Chin D, Richter King C and Huberman E (1990) Differentiation of cultured human breast cancer cells (AU-565 and MCF-7) associated with loss of cell surface *HER-2/neu* antigen. *Mol Carcinogen* **3**: 350–362

Benz CC, Scott GK, Sarup JC, Johnson RM, Tripathy D, Coronado E, Shepard HM and Osborne CK (1993) Estrogen-dependent, tamoxifen-resistant tumorigenic growth of MCF-7 cells transfected with HER2/neu. *Breast Cancer Res Treat* **24**: 85–95

Bissonnette RP, Echeverri F, Mahboubi A and Green DR (1992) Apoptotic cell death induced by c-*myc* is inhibited by *bcl*-2. *Nature* **359**: 552–554

Bollag W, Majewski S and Jablonska S (1994) Cancer combination chemotherapy with retinoids: experimental rationale. *Leukemia* **8** (suppl.3): S11–S15

Caliaro MJ, Vitaux P, Lafon C, Lochon I, Néhmé A, Valette A, Canal P, Bugat R and Jozan S (1997) Multifactorial mechanism for the potentiation of cisplatin (CDDP) cytotoxicity by all-*trans* retinoic acid (ATRA) in human ovarian carcinoma cell lines. *Br J Cancer* **75**: 333–340

Costa A, De Palo G, Decensi A, Formelli F, Chiesa F, Nava M, Camerini T, Marubini E and Veronesi U (1995) Retinoids in cancer chemoprevention. Clinical trials with the synthetic analogue fenretinide. *Ann NY Acad Sci* **768**: 148–162

D'Souza B, Berdichevsky F, Kyprianou N and Taylor-Papadimitriou J (1993) Collagen-induced morphogenesis and expression of the $\alpha_2$-integrin subunit is inhibited in c-*erb*B-2-transfected human mammary epithelial cells. *Oncogene* **8**: 1797–1806

De Bortoli M, Dati C, Antoniotti S, Maggiora P and Sapei L (1992) Hormonal regulation of c-*erb*B-2 oncogene expression in breast cancer cells. *J Steroid Biochem Mol Biol* **43**: 21–25

Fanjul A, Dawson MI, Hobbs PD, Jong L, Cameron JF, Harlev E, Graupner G, Lu X-P and Pfahl M (1994) A new class of retinoids with selective inhibition of AP-1 inhibits proliferation. *Nature* **372**: 107–111

Fanjul AN, Delia D, Pierotti MA, Rideout D, Yu J-Q and Pfahl M (1996) 4-Hydroxyphenyl retinamide is a highly selective activator of retinoid receptors. *J Biol Chem* **271**: 22441–22446

Fenaux P, Chomienne C and Degos L (1997) Acute promyelocytic leukemia: biology and treatment. *Semin Oncol* **24**: 92–102

Flicker SH, Schneider S, Offterdinger M, Dittrich E, Fazeny B, Valenta R, Huber H, Dittrich Ch and Grunt ThW (1997) Tyrosine kinase signaling pathways control the expression of retinoic acid receptor-α in SK-BR-3 breast cancer cells. *Cancer Lett* **115**: 63–72

Formelli F and Cleris L (1993) Synthetic retinoid fenretinide is effective against a human ovarian carcinoma xenograft and potentiates cisplatin activity. *Cancer Res* **53**: 5374–5376

Grunt ThW and Huber H (1994) The family of c-erbB genes: from basic research to clinical oncology. *Onkologie* **17**: 346–357

Grunt ThW, Somay C, Pavelka M, Ellinger A, Dittrich E and Dittrich Ch (1991) The effects of dimethyl sulfoxide and retinoic acid on the cell growth and the phenotype of ovarian cancer cells. *J Cell Sci* **100**: 657–666

Grunt ThW, Somay C, Oeller H, Dittrich E and Dittrich Ch (1992*a*) Comparative analysis of the effects of dimethyl sulfoxide and retinoic acid on the antigenic pattern of human ovarian adenocarcinoma cells. *J Cell Sci* **103**: 501–509

Grunt ThW, Somay C, Ellinger A, Pavelka M, Dittrich E and Dittrich Ch (1992*b*) The differential effects of transforming growth factor-α1 and N,N-dimethylformamide on proliferation and differentiation of a human ovarian cancer cell line (HOC-7). *J Cell Physiol* **151**: 13–22

Grunt ThW, Oeller H, Somay C and Dittrich Ch (1993) Different propensity for spontaneous differentiation of cell clones isolated from the ovarian surface epithelial cell line HOC-7. *Differentiation* **53**: 45–50

Grunt ThW, Saceda M, Martin M-B, Lupu R, Dittrich E, Krupitza G, Harant H, Huber H and Dittrich Ch (1995) Bidirectional interactions between the estrogen receptor and the c-erbB-2 signaling pathways: heregulin inhibits estrogenic effects in breast cancer cells. *Int J Cancer* **63**: 560–567

Hancock MC, Langton BC, Chan T, Toy P, Monahan JJ, Mischak RP and Shawver LK (1991) A monoclonal antibody against the c-*erb*B-2 protein enhances the cytotoxicity of *cis*-diaminedichloroplatinum against human breast and ovarian tumor cell lines. *Cancer Res* **51**: 4575–4580

Hynes NE, Gerber HA, Saurer S and Groner B (1989) Overexpression of the c-erbB-2 protein in human breast tumor cell lines. *J Cell Biochem* **39**: 167–173

Kalthoff H, Roeder Ch, Gieseking J, Humburg I and Schmiegel W (1993) Inverse regulation of human ERBB2 and epidermal growth factor receptors by tumor necrosis factor α. *Proc Natl Acad Sci USA* **90**: 8972–8976

Kazmi SMI, Plante RK, Visconti V and Lau CY (1996) Comparison of *N*-(4-hydroxyphenyl)retinamide and all-*trans*-retinoic acid in the regulation of retinoid receptor-mediated gene expression in human breast cancer cell lines. *Cancer Res* **56**: 1056–1062

Krupitza G, Hulla W, Harant H, Dittrich E, Kallay E, Huber H, Grunt T and Dittrich C (1995) Retinoic acid induced death of ovarian carcinoma cells correlates with c-myc stimulation. *Int J Cancer* **61**: 649–657

 © Cancer Research Campaign 1998

Marth C, Cronauer MV, Doppler W, Öfner D, Ullrich A and Daxenbichler G (1992) Effects of interferons on the expression of the proto-oncogene HER-2 in human ovarian carcinoma cells. *Int J Cancer* **50**: 64–68

Martin SJ, Reutelingsperger CPM, McGahon AJ, Rader JA, van Schie RCAA, LaFace DM and Green DR (1995) Early redistribution of plasma membrane phosphatidylserine is a general feature of apoptosis regardless of the initiating stimulus: inhibition by overexpression of Bcl-2 and Abl. *J Exp Med* **182**: 1545–1556

Mroczkowski B, Mosig G and Cohen S (1984) ATP-stimulated interaction between epidermal growth factor receptor and supercoiled DNA. *Nature* **309**: 270–273

Nehme A, Albin N, Caliaro MJ, Guichard S, Jozan S, Julia A-M, Bugat R and Canal P (1995) Mechanism of interaction between cisplatin and human recombinant interferon gamma in human ovarian cancer cell lines. *Int J Cancer* **61**: 643–648

Nugent A, McDermott E, Duffy K, O' Higgins N, Fennelly JJ and Duffy MJ (1992) Enzyme-linked immunosorbent assay of c-*erb*B-2 oncoprotein in breast cancer. *Clin Chem* **38**: 1471–1474

Paik S (1992) Clinical significance of c-erbB-2 (HER-2/neu) protein. *Cancer Invest* **10**: 575–579

Pellegrini R, Mariotti A, Tagliabue E, Bressan R, Bunone G, Coradini D, Della Valle G, Formelli F, Cleris L, Radice P, Pierotti MA, Colnaghi MI and Ménard S (1995) Modulation of markers associated with tumor aggressiveness in human breast cancer cell lines by *N*-(4-hydroxyphenyl)retinamide. *Cell Growth Diff* **6**: 863–869

Pietras RJ, Fendly BM, Chazin VR, Pegram MD, Howell SB and Slamon DJ (1994) Antibody to HER-2/*neu* receptor blocks DNA repair after cisplatin in human breast and ovarian cancer cells. *Oncogene* **9**: 1829–1838

Read LD, Keith DJr, Slamon DJ and Katzenellenbogen BS (1990) Hormonal modulation of HER-2/neu protooncogene messenger ribonucleic acid and p185 protein expression in human breast cancer cell lines. *Cancer Res* **50**: 3947–3951

Rustin G (1994) Synopsis of workshop on treatment of solid tumors. *Leukemia* **8** (suppl. 3): S85–S86

Saceda M, Grunt ThW, Colomer R, Lippman ME, Lupu R and Martin MB (1996) Regulation of estrogen receptor concentration and activity by an erbB/HER ligand in breast carcinoma cell lines. *Endocrinology* **137**: 4322–4330

Sacks PG, Harris D and Chou T-C (1995) Modulation of growth and proliferation in squamous cell carcinoma by retinoic acid: a rationale for combination therapy with chemotherapeutic agents. *Int J Cancer* **61**: 409–415

Shalinsky DR, Bischoff ED, Gregory ML, Thomazy V, Lamph WW, Heyman RA, Davies PJA and Hayes JS (1995) Antitumor efficacy of LGD 1057 (9-*cis* retinoic acid) in combination with cisplatin (DDP) in human head and neck xenografts. *Proc Am Assoc Cancer Res* **36**: 296 (abstract 1762)

Somay C, Grunt ThW, Mannhalter Ch and Dittrich Ch (1992) Relationship of myc protein expression to the phenotype and to the growth potential of HOC-7 ovarian cancer cells. *Br J Cancer* **66**: 93–98

Tang CK, Perez C, Grunt T, Waibel C, Cho C and Lupu R (1996) Involvement of heregulin-β2 in the acquisition of the hormone-independent phenotype of breast cancer cells. *Cancer Res* **56**: 3350–3358

Trauth BC, Klas C, Peters AM, Matzku S, Moller P, Falk W, Debatin KM and Krammer PH (1989) Monoclonal antibody-mediated tumor regression by induction of apoptosis. *Science* **245**: 301–305

Tsai C-M, Chang K-T, Perng R-P, Mitsudomi T, Chen M-H, Kadoyama C and Gazdar AF (1993) Correlation of intrinsic chemoresistance of non-small-cell lung cancer cell lines with HER-2/neu gene expression but not with ras gene mutations. *J Natl Cancer Inst* **85**: 897–901

Veronesi U, De Palo G, Costa A, Formelli F and Decensi A (1996) Chemoprevention of breast cancer with fenretinide. *IARC Sci Publ* **136**: 87–94

Yu D, Liu B, Tan M, Li J, Wang S-S and Hung M-C (1996) Overexpression of c-*erb*B-2/*neu* in breast cancer cells confers increased resistance to taxol via *mdr-1*-independent mechanisms. *Oncogene* **13**: 1359–1365

© Cancer Research Campaign 1998